# How to adapt sexual and reproductive health services to the needs and circumstances of trans people— a qualitative study in Colombia

**DOI:** 10.1186/s12939-020-01250-z

**Published:** 2020-10-26

**Authors:** Mariana Calderón-Jaramillo, Ángel Mendoza, Natalia Acevedo, Luz Janeth Forero-Martínez, Sandra Marcela Sánchez, Juan Carlos Rivillas-García

**Affiliations:** 1 Asociación Profamilia, Bogotá, D.C. Colombia; 2 Pittsburg University, Pittsburg, USA; 3 Sociology and political institutions, Bogotá, Colombia

**Keywords:** Health equity, Reproductive Health services, Sexual Health; sexual and gender minorities, Delivery of healthcare

## Abstract

**Background:**

People living a trans-life require access to equitable healthcare services, policies and research that address their needs. However, trans people have experienced different forms of violence, discrimination, stigma, and unfair access barriers when dealing with healthcare providers. Therefore, adapting sexual and reproductive health services with the purpose of providing more equitable, inclusive and discrimination-free healthcare services is an urgent need. The article presents an example of how operative research can be used in order to adjust sexual and reproductive healthcare services to trans people’s needs, identities and circumstances.

**Methods:**

This is a qualitative study written from a constructivist perspective, and it is based on the voices and experiences of trans people in four major cities in Colombia. The research used a combination of focus groups of discussion (*n* = 6) and in-depth interviews with trans people (*n* = 13) in Barranquilla, Bogota, Cali and Medellin. This research had two specific objectives: i) identifying the main sexual and reproductive health needs of people living a trans-life; and ii) generating new evidence in order to guide the adaptation of sexual and reproductive health services centered to trans people’s needs, identities, and circumstances. Qualitative data codification and analysis was using NVivo.

**Results:**

Once access barriers to sexual and reproductive health services, unmet sexual and reproductive health needs were identified, the research helped define strategies to adapt sexual and reproductive health services to the needs, identities, and circumstances of people living a trans-life in Colombia. Amongst the main barriers found were healthcare costs, lack of insurance, stigmatization, discrimination and abuse by health care providers. Perhaps among the most notable sexual and reproductive health needs presented were trans-specific services such as sensitive assistance for the transition process, endocrinology appointments, and sex reaffirmation surgeries.

**Conclusions:**

The evidence obtained from this research allowed Profamilia, a Colombian healthcare provider, to adapt the sexual and reproductive health services it provides to people living a trans-life in Colombia. Furthermore, it was possible for Profamilia to design and implement an inclusive sexual and reproductive health program that specifically addresses trans people’s needs, identities, and circumstances.

## Background

Trans people require policies and research that are in line with their needs, as well as access to unbiased healthcare services [1, 2]. For this reason, sexual and reproductive health services must be modified with the aim of increasing access to more equitable, inclusive and discrimination-free healthcare services for trans people [1]. in order to achieve that goal, healthcare providers have to identify unmet sexual and reproductive health needs; to acknowledge the diversity of social subjects who require services that are fitting to their identities; and to recognize the actions of social determinants of health on specific contexts in which trans people need access to health.

This can undoubtedly contribute to toward the accomplishment of the third Sustainable Development Goal [3], in particular objective 3.7: “By 2030, ensure universal access to sexual and reproductive health-care services, including for family planning, information and education, and the integration of reproductive health into national strategies and programmes”. The World Health Organization (WHO) encourages countries to safeguard sexual and reproductive rights through healthcare services centered around the needs, identities, and circumstances of people and discourages them from leaving anyone behind [1].

Trans people have historically faced different types of violence, discrimination, stigma, and unfair access barriers to healthcare services at all levels of the healthcare system, as well as social exclusion from information systems, healthcare programs, and legal frameworks. Amongst the most common healthcare barriers found by different studies are the lack of healthcare coverage; denial of essential services such as sexual and reproductive health services; lack of knowledge and training of healthcare staff, social prejudice and imposition of either masculinity or femininity by healthcare providers depending on the patient’s assigned sex at birth [4–11].

The category “trans” emerged on the twenty-first century under a regular practice of medicine based on two sexes [12, 13]. This category makes reference to the experiences of those people who, throughout their life decide to transform the sex that was assigned to them at birth (transsexual) using as reference a definition of sex based on genital and hormonal aspects [14]; this category is also used for people who decide to transform their gender from the one they built during their socialization development which is commonly based on the sex that was assigned to them at birth (transgender). Although both categories allow some practices, decisions, and transformations that are part of the transition processes to be distinguished, the category trans works as an umbrella [15, 16] and encompasses a broad spectrum of trans-life experiences.

Colombia has kept no records of trans-people that would allow for their socio-demographic characterization. The National Demographic and Health Survey (ENDS) of 2015 included the gender identity variable [17]. However, it was only filled out in 36 cases. Additionally, most administrative records do not allow for the disaggregation of the information by trans people, thus holding a mantle of invisibility in information systems, which prevent getting to know their needs, contexts, and circumstances in greater detail, and thus limit the ability to address their needs accordingly.

According to the Exploratory Survey of Trans People who have used healthcare services in Colombia [18], 85% think that healthcare providers are not sufficiently educated in regards to their needs; 83% consider that health care professionals do not possess enough training and skills; 69% think that healthcare services do not provide them with inclusive care, and; 57% have forgone the use of health services out of fear that their gender (trans condition) will affect their medical care.

Prior research demonstrates how trans people have been excluded from sexual and reproductive health services and outline the fact that they have been exposed to different forms of violence by healthcare service providers. There is broad evidence on the existence of multiple access barriers that generate various forms of violence, discrimination, and stigmatization towards trans people who seek healthcare services [4, 6–11, 19]. These barriers have systematically affected trans people. As a result, people have been coerced to perform hormone treatment on their own or perform non-medical body interventions that put their lives at risk or make them prone to suffering from long-term negative effects [16, 19].

Nevertheless, to our knowledge, a limited number of studies have addressed this issue on how to tailor sexual and reproductive health services to trans populations and to eliminate medical barriers for equitable care. The evidence that is available is seriously limited in the context of Latin America and the Caribbean [2]; countries such as Brazil, Mexico [20], Argentina [21] and also the USA [22] and Spain [23], have implemented responses to the needs of trans people, but lean towards a pathologizing view. From a qualitative perspective, this study was oriented by the following research questions: How have trans people experienced access to sexual and reproductive healthcare services? Which have been the barriers they have faced? and also: What are their expectations around overcoming these barriers?

The goal of this research was twofold: first, to identify sexual and reproductive health services access barriers and needs experienced by trans people. Second, to describe strategies to adapt sexual and reproductive health services to the needs, identities and circumstances of trans people, with the purpose to contribute for the fulfillment of universal sexual and reproductive health coverage of trans people as per the 2030 agenda.

## Methods

### Study design

This was a qualitative study performed under a constructivist perspective and was based on the voices and experiences of trans people in four major cities in Colombia. The research was focused on identifying barriers from what trans people shared about their encounters with sexual and reproductive healthcare services, as well as their expectations for what aspects should be included in these services in order to meet their specific needs. This methodology was formulated with the aim of hearing trans people’s views, and thus guarantee that the process of service adaptation was aligned with those who the services are oriented to.

### Target population and data collection

The process of information collection was conducted in four Profamilia clinics located in Barranquilla, Bogota, Cali, and Medellin. Profamilia is a non-profit sexual and reproductive healthcare provider which has been committed to uphold the rights of Colombians for the past 55 years. The selection criterion of the cities was based on the degree of acceptance of the LGBTI population: High acceptance (Cali), medium acceptance (Medellin and Bogota) and low acceptance (Barranquilla) based on the results of the National Demographic and Health Survey (ENDS) [17]. It is important to mention that during the field research, some trans organizations in each city came to be part of the process, aiming to ensure the participation of trans people. The research team was composed by interdisciplinary members with the intention of integrating different levels of expertise, perspectives, and experiences. It is important to highlight that one of the main researchers identifies as trans, and this gave strength and self-reflection to the approach, the findings, and the discussions.

### Focus groups of discussion and semi-structured interviews

The fieldwork consisted in conducting focus groups of discussion and semi-structured interviews. The number of participants in the research per city is described as follows: Barranquilla (*n* = 12), Bogotá (*n* = 23), Cali (*n* = 17), and Medellín (*n* = 15). In total, 5 focus groups of discussion were carried out with a total of 54 participants; on average 10 people participated in each group. In Bogotá, two focus groups were conducted due to the strength of trans community, while in the remaining cities only one was held; additionally, 13 interviews were made, three in each one of the cities with the exception of Cali where one more interview was conducted due to saturation criteria.

The focus groups of discussion and semi-structured interviews were conducted by a qualitative research assistant of the team who received training on the research and the application of a common guide in the field. Participants’ selection and recruitment process was made through snowball technique, a non-probability sampling method in coordination with trans organizations and networks in the four cities. The sample was subject to saturation criteria: in the interviews this happened when the research team stopped identifying differences among the experiences of trans people regarding sexual and reproductive health services; whereas in the case of focus groups of discussion, saturation was the result of iteration in respect to the barriers in healthcare services that participants had experienced and the meanings of people-centered care. All focus groups were conducted in Spanish and all participants agreed being audio-recorded during the discussion. Immediately afterwards, observational field notes were taken.

The focus group discussions helped identify barriers and facilitating factors in regards to the access of sexual and reproductive health services, as well as the exploration of the meaning that the community gave to the idea of people-centered medical attention; the focus groups aimed to compare different experiences and build consensus about some issues, particularly surrounding common barriers and expectations. Furthermore, the interviews allowed researchers to delve deeper into specific encounters trans people had with sexual and reproductive health services and their overall relations with providers. During these stories of specific situations, the interviewees shared revealed a complex panorama.

### Data analysis

Semi-structured interviews and focus groups of discussion were recorded, transcribed and processed using the NVivo software. The coding process was done by setting topics and categories beforehand, although it was also open to emerging ones. The main coding topics were selected from reviewing previous literature and also were used to structure interviews and focus groups guides. The topics were: i) sexual and reproductive health needs; ii) services access barriers; and iii) strategies to adapt services to the needs, identities, and circumstances of trans people. Within each one of these topics, emerging categories were the result of specific ways or conceptual refining of topics meaning. Research results were discussed among the authors and presented to Profamilia with the goal of promoting a reform of sexual and reproductive services.

### Ethical and gender considerations

The voluntary participation of people in the research was obtained through prior informed consent. In the study, people over 18 years of age that identified themselves as trans were included. The research guaranteed equal participation for both trans women and men in focus groups of discussion and interviews. All the information was anonymized to avoid the identity of participants. This research received ethical approval on the 24th of August, 2018 from the Ethical committee for Research in Profamilia (CEIP by its name in Spanish) through CEIP-201808.

## Results

The results are presented in the following order: i) sexual and reproductive health services access barriers experienced by trans people; ii) sexual and reproductive health needs; and iii) strategies to adapt sexual and reproductive health services to the needs, identities and circumstances of trans people.

### Sexual and reproductive health services access barriers

The information analysis allowed for the identification of barriers and their effects. The first topic that came up is linked to the out-of-pocket expenses for essential sexual and reproductive health services. This out-of-pocket spending is often the main access barrier to healthcare services and is linked to other access barriers such as i) lack of health insurance; ii) red tape at all levels of the healthcare system; iii) discomfort in the interaction with healthcare staff (due to bias) iv) denial of medical attention by healthcare providers; v) lack of knowledge about gender identity; vi) pathologizing trans identities; vii) pressure to fit into sexual binarism and vii) previous experiences of violence and abuse by healthcare providers.

These barriers are not only related to the cost of services, the prejudices and stigmatization toward trans people healthcare service providers., But also to the limitation of effective access at the time and places at which they may be required. Among all the participants, there was consensus around the fact that the perceptions and negative opinions that exist about trans people and the barriers to health care services have been normalized. For trans people, accordingly, this has been translated into a deep mistrust towards healthcare service providers each time that their identities are not understood or respected. The following quote expresses that sentiment:

It’s like there’s only men and women, You know what I mean? And then there is only treatment for those people. But when we get there, it’s like “something new is coming” I feel that it shouldn’t be like that, I mean, they should understand that there is a human diversity in the world and that they should treat us all the same

*(Focus group participant - Bogota)*

This testimony signals the discontent that certain barriers generate. Within focus groups of discussion, research participants also indicated that the lack of sensitivity translated to mistrust and frustration about healthcare services.

### Sexual and reproductive needs

In regards to sexual and reproductive health necessities for people living a trans-life, a consensus was reached on the existence of two dimensions: i) general sexual and reproductive health necessities, and ii) trans-specific sexual and reproductive health necessities.

General sexual and reproductive health necessities include: i) birth control and ob/gyn care; ii) attention for cases of gender-based violence; iii) abortion and post-abortion services; iv) access to mammography, hysterectomy and transvaginal ultrasound procedures; v) sexologist, gynecologist, urologist, contraception and fertilization visits; vi) comprehensive sexual education; vii) mental health and psychosocial support services; and vii) STDs care and treatment. One participant said that the type of care he expected from healthcare services regarding these topics has a lot to do with getting equal treatment.

I have always said this: “we don’t need any special treatment because we are normal human beings”, we just need human treatment, we just need them to treat us like they treat everyone else.

(Interview with a trans person in Barranquilla)

On the other hand, trans people participating in this research also highlighted specific necessities, also known as trans-specific needs, which include: i) sensitive advice and guidance for the beginning and development stages of their transition; ii) visits to endocrinologists; iii) sex reassignment surgeries and iv) a comprehensive care packages. The field research allowed us to interpret what each of these services means for trans people.

#### Trans sensitive care for the beginning and development of their transition

Trans people who participated in the research recognize that their transition is an important and complex process, for which many times they require psychological assistance. The acknowledgement of this mental and psychological dimension of the transition brings the attention to the need for sensitive advice and guidance throughout the beginning and transitional phases of their transformation journeys by means of avoiding pathologizing trans people:

“The transition is not only physical; the transition is also mental”

(Focus group participant in Cali).

#### Visit to endocrinologists

For most trans people, their transition includes hormone therapy, which is normally one of the first interventions on their bodies. Experiencing access barriers to high-quality healthcare services leads trans people to self-medicate. Also, the lack of skills and training from the healthcare staff make people question the medical advice and recommendations that they are given:

“And then we ask them about the feminization processes or hormone replacement therapy and all that. At the end of the day, we know more about this stuff than they [healthcare providers] do”

(Focus group participant, Bogotá).

#### Sex reassignment surgery

Although not all transitions include sex reassignment surgery, for many people this procedure is a fundamental part of the process. When this topic was discussed during focus groups of discussion, discussions took place on whether the need to get this surgery existed. These positions are indicators of the multiple forms of transition that there are, however, the multiplicity of the issue is limited by the huge difficulties in access to such services and their effects on the physical integrity of people. An example of this disagreement emerged during a focus group of discussion in Medellin in which one of the participants asked “Who would not love to have a sex reassignment surgery?” to which another participant replied “that [would take] the essence of being trans away from us”. In this specific discussion emerged different positions about who is a trans persons and which are the physical transformation they should go through.

#### Comprehensive care process for trans people

The specific character of many of the sexual and reproductive health needs trans people have, as well as the different medical attention barriers that they face points to the need of a comprehensive care package that can reshape sexual and reproductive health services and achieve financial protection for trans people. There was strong consensus among the participants in regards to the need for healthcare providers to define a service package that can address all the specific needs of trans people.

Both in the interviews and the focus groups of discussion there was evidence that the participants are interested in counting on the assistance from the healthcare staff during their transition. This requires a step forward in sexual and reproductive health development and training that is gender-sensitive, in particular for trans people.

*[…] I think that in the trans people issue, proper attention would be to be called by your name, for your identity to be respected, for them to know that […] there are trans people that also want to have kids, for them to guide us throughout that process […]*

*(Focus group participant, Medellín)*

### Strategies to adjust sexual and reproductive health services

Also, from the data analysis of the focus groups of discussion and semi-structured interviews emerged diverse result of specific ways or strategies which allowed understand the needs and challenges to adjust sexual and reproductive health services. Some strategies through which health services can be enhanced and adapted to the needs, identities, and circumstances of people living a trans-life were identified. To get to know and build these strategies, inquiries were made about the expectations that trans people had at the time of seeking sexual and reproductive health services. This type of people-centered healthcare attention strategies suggests that trans people should be a sexual and reproductive health priority. Trans people should have effective access to healthcare services and to achieve so, the technical skills of staff and healthcare providers must be strengthened. This includes gender-sensitive approaches at the moment of addressing the needs of trans people and ultimately, an inclusive, safe, dignified and discrimination and stigma-free sexual and reproductive health attention. In other words, a differential approach is required to guarantee sexual and reproductive rights.

Table 1 synthesizes the strategies that can be implemented as a response to the previously described sexual and reproductive health access barriers. Column A presents the health care attention barriers and Column B, the identified strategies to respond to the necessities, identities, and circumstances of trans people.

**Table 1:**
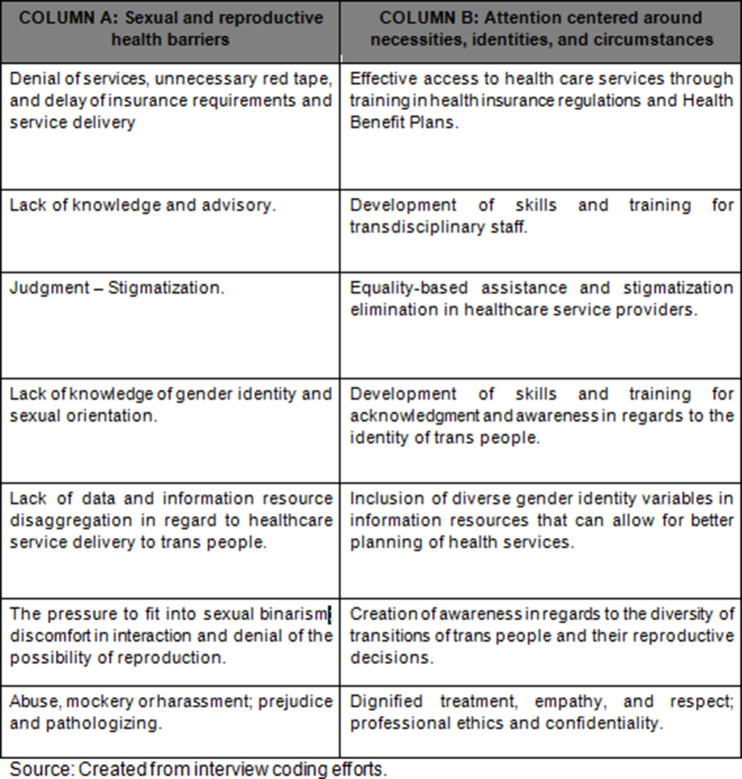
Sexual and reproductive health access barriers and strategies for sexual and reproductive health attention from a differential approach

## Discussion

Throughout their whole lives, trans people face different access barriers to health care services in general, and especially to sexual and reproductive health services. Such barriers are the result of different social health determining factors. For example at the structural level, the different social rules, stigmatization, discrimination, and transphobia position trans people at social disadvantage, which results in inequality in access to essential sexual and reproductive health services. On the other hand, at the individual and intrapersonal levels, it is their support networks, the specific way in which they want to experience their transition, and the context in which they look for health services and attention the factors which determine their access and attention to sexual and reproductive health.

The access barriers to sexual and reproductive health services identified by this research match the existing evidence. Different types of research on this issue around the world [2, 4, 7–11] and in Colombia [6, 15, 16, 19] have caught a lot of attention. However, time and time again the documentation and production of evidence regarding the barriers and complaints of trans people have remained unadopted within healthcare services and processes. Along these lines, the necessity of applying participative methodologies guided towards the adaptation and improvement of services has been pointed out [4].

It is necessary to keep researching this subject; the qualitative approaches have to be complemented with socio-demographic and epidemiologic quantitative analyses that shed light on the magnitude of this issue and help uncover its effects on the health of trans people who face these healthcare services access barriers. Additionally, it is necessary to create new research that can capture other intersections that affect the needs of trans people according to their age and their given stage in life, their race or ethnic group, disability, geographic location, among others.

The newly generated evidence aligns with this purpose for two reasons: first, attention barriers are not the only focus— adaptation strategies and needs in sexual and reproductive health were also expressed by trans people; secondly, since this is an operational research, one of the objectives was for the evidence to effectively and safely translate into sexual and reproductive health services that are based on the realities of trans people. By centering the attention on the narratives of trans people, we wanted to guarantee the adaptation of healthcare services according to their needs, identities, and circumstances, as well as questioning research methodologies that have historically labeled trans people as a study and intervention subject and not as agents of transformation.

This study has some limitations that are worth mentioning. First, the results are limited to the four cities in which the implementation of the project took place; secondly, although we sought the participation of trans people who represented different contexts and backgrounds, most of the participants belonged to trans organizations or LGBTI groups. Thus, we must ask ourselves whether trans people who have not been as close to social LGBTI movements have the same necessities and expectations.

The evidence obtained from this research allowed Profamilia to adapt its sexual and reproductive health services and cater to trans people. Based on the results, it was possible to design and implement a sexual and reproductive health attention program for trans people based on three principles: (i) de-pathologizing trans-life experiences; (ii) centering care around the needs and circumstances of people and (iii) advising sensitively throughout transition under informed consent as the articulating axis that fosters body, sexual and gender autonomy, right to life and free development of personality.

## Conclusions

Thinking about the needs, identities, and circumstances of trans people as they pertain to health and especially to sexual and reproductive health includes questioning the existing sexual and gender binarism attention parameters and the medical criteria that up to now seemed unwilling to change. This gives way for healthcare service providers to go from regulating and normalizers of sexual and gender identities, to becoming caring assistants during transition processes. Accordingly, healthcare providers must begin to see that users are self-determining in regard to their sexuality and identity, and must recognize their body autonomy; a more horizontal relation between users and healthcare providers must be established.

The adaptation of the sexual and reproductive health services to trans people’s needs has not ended; it is not enough to have technical clinical knowledge. The transformation of health services must be a continuous process that has to involve the multiplicity of trans-life experiences and the overall diversity of people in all the aspects of their life. The process must consider that these are services that have to be continuously reviewed and updated. In other words, these are services that have to change over time and that will have to change along with people.

The research performed gave way to the establishment of strategies for the construction of a healthcare attention program for trans people. Such strategies finally became the roadmap that allowed us to organize our internal structure. Below, we will list such strategies, hoping that they can be useful for other health actors that wish to adjust their healthcare services:

1. Community incidence strategies: to improve and invest in the strengthening of trust amongst trans people.
2. Awareness and internal training processes: to eliminate violence and discrimination in the health service delivery through awareness campaigns.
3. Technical training for healthcare professionals: to develop high-quality technical and training skills in the clinical health approach.
4. Building an interdisciplinary team: to create paths, protocols and sexual and reproductive healthcare programs for trans people.
5. Research: to ensure knowledge management, dissemination and communication of this practice with other service providers and government sectors.
6. External advocacy: to generate political incidence processes than can promote the protection of the rights of trans people.

The construction of this model is a historical debt owed to trans people, one that may provide them with access to health services in a dignified and safe way which is cognizant of their needs. Overall, it is very important to boost our country scientifically, academically and culturally to move forward in the promotion of rights for all people.

## Data Availability

Qualitative study, the data is restricted to the authors to guarantee confidentiality.

## Abbreviations

ENDS: Encuesta Nacional de Demografía y Salud (National Demographic and Health Survey)
STD: Sexually Transmitted Diseases
LGBTI: Lesbian, Gay, Bisexual, Trans, Intersexual
WHO: World Health Organization
